# Combretastatins D series and analogues: from isolation, synthetic challenges and biological activities

**DOI:** 10.3762/bjoc.19.31

**Published:** 2023-03-29

**Authors:** Jorge de Lima Neto, Paulo Henrique Menezes

**Affiliations:** 1 Universidade Federal de Pernambuco, Departamento de Química Fundamental, Recife-PE, 50740-560, Brazil

**Keywords:** combretastatin D, corniculatolide, isocorniculatolide, macrocycles

## Abstract

The combretastatin D series and its analogues, corniculatolides and isocorniculatolides belong to a class of macrocycles called cyclic diaryl ether heptanoids (DAEH). This review is intended to highlight the structure elucidation, biosynthesis, and biological activity of these compounds as well as the use of different strategies for their synthesis.

## Introduction

Conceptually, cyclic molecules containing more than 12 covalently connected atoms are called macrocycles and this class comprises several structurally distinct compounds [1]. Due to their structural characteristics and their different biological activities, the isolation of new macrocycles and their synthesis finds several examples described in the literature and has already been the subject of review articles [2–4].

More recently, cyclic diaryl ether heptanoids (DAEH) [5], another class of macrocycles has been attracting attention not only because of their structural and stereochemical characteristics, but also for shown biological activities, with potential therapeutic application [6–12]. Examples of this class of compounds are the combretastatin D series [13] and their analogues, corniculatolides and isocorniculatolides [14], which are structurally characterized as an oxa[1.7]*meta-para*cyclophane framework.

**Figure 1:**
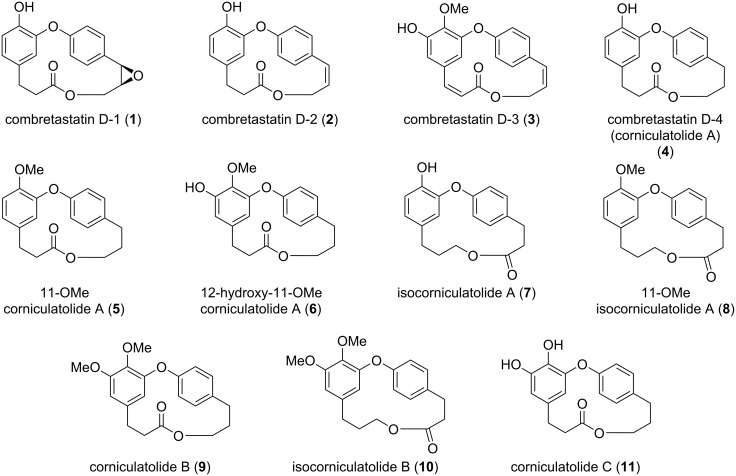
Structures of some members of the combretastatin D series, corniculatolides, and isocorniculatolides.

In the literature we can find reports about the isolation/synthesis of combretastatins D and their analogues which showed different biological activities, e.g., antineoplastic, anti-inflammatory, and α-glucosidase inhibition [13–15]. The presence or absence of certain functional groups in the structure of these compounds, such as a *cis* double bond or the position of a hydroxy or methoxy group play a crucial role in their biological activity as will be shown later in this review (Figure 1).

Although there are reviews dealing with macrocyclic compounds available in the literature, there is none which focuses only on combretastatins D and their isomers. Therefore, this review is divided into three main parts: the first comprises the isolation of these compounds from natural sources. In the following part, the biosynthetic pathway and the total/formal synthesis of these compounds will be summarized and, finally, the biological activity and potential for therapeutic applications will be addressed. Thus, this review demonstrates not only the challenge in isolating these compounds, but also the synthetic complexity of preparing them in sufficient quantities for the evaluation of biological/pharmacological activities.

## Review

### 1 Isolation

#### 1.1 Isolation of combretastatins D series

Combretastatins comprise a large family of structurally diverse natural products divided into the “A” (*cis*-stilbenes), “B” (dehydrostilbenes), “C” (phenanthrenes), and “D” (macrocyclic diaryl ethers) series found in plants present on the African and Asian continent [15].

The first report of this class of compounds was made by Pettit and co-workers when they isolated combretastatin D-1 (**1**) from a CH_2_Cl_2_/MeOH extract of *Combretum caffrum*, a South African tree [16]. From 77 kg of stem wood, a fraction was obtained using a Sephadex LH-20 column by partition chromatography to afford two active fractions. One of the fractions was chromatographed on a silica gel column to give compound **1** (180 mg). The other fraction was again chromatographed on a Sephadex LH-20 column and the resulting active fraction was chromatographed on a silica gel column to afford a new fraction. Re-chromatography in a silica gel column using gradient elution afforded combretastatin D-2 (**2**, 5.8 mg) [17].

The general structures of combretastatins D-1 (**1**) and D-2 (**2**) were established by Pettit and Singh [16–17] by analysis of NMR and mass spectra and confirmed by X-ray crystallography in an initial report. However, attempts to determine the absolute configuration of the epoxide present in compound **1** based on crystallographic data were unsuccessful. By matching the sign of the Cotton effect curves obtained in the combretastatin D-1 spectrum with the appropriate chiral epoxides, the authors assigned the absolute stereochemistry of the epoxide ring as 3*R*,4*S*. This attribution was controversial and was only definitively established years later, as will be shown in this review.

In 2005, Vongvanich and co-workers isolated combretastatins D-3 (**3**) and D-4 (**4**) from *Getonia floribunda*, a woody climber commonly found in many areas of Thailand [18]. From 3 kg of dried stems of *Getonia floribunda* macerated in CH_2_Cl_2_ it was obtained a crude extract which was chromatographed using Sephadex LH-20. The obtained fractions were re-chromatographed on Sephadex LH-20 and the obtained fractions were further purified by silica gel column chromatography. One of the obtained fractions contained pure compound **3** (10.6 mg), while another fraction was further purified by silica gel column chromatography to give compound **4** (6.8 mg).

#### 1.2 Isolation of corniculatolides and isocorniculatolides

Corniculatolides and isocorniculatolides, isomeric macrolides of combretastatins D, were isolated by Ponnapalli’s group from two distinct species of trees. From the bark of *Aegiceras corniculatum*, the authors isolated the known compound **4** and the isomeric corniculatolides. The isolation of the compounds was achieved from 5 kg of air-dried bark of the aforementioned tree, which was grounded and then extracted with CHCl_3_ using a Soxhlet apparatus, furnishing 32.0 g of the crude extract. This extract was subjected to a vacuum liquid chromatography on silica gel to yield several fractions. Some of them were tested and the ones that exhibited antimicrobial activity were further fractionated on silica gel chromatographic column eluted with hexane and acetone (8:2) to yield four different fractions. The fractions comprised 11-*O*-methylcorniculatolide A (**5**, 10 mg), 12-hydroxy-11-*O*-methylcorniculatolide A (**6**, 1 mg) and 11-*O*-methylisocorniculatolide A (**8**, 2 mg). The authors had some difficulties to isolate isocorniculatolide A (**5**) using chromatography, however, it was obtained by crystallization from hexane and acetone (4 mg). The same strategy was used to isolate the known compound corniculatolide A (**4**, 10 mg) as colorless crystals [14].

Later the same group reported the isolation of corniculatolide B (**9**), isocorniculatolide B (**10**), and corniculatolide C (**11**) from *Xylocarpus granatum*, a tree commonly found in Southeast Asia and along the Indian Ocean coastline. The air-dried stems of *X. granatum* (5 kg) were powdered and extracted successively within hexane, CHCl_3_, and acetone in a Soxhlet apparatus. The crude CHCl_3_ extract (21 g) was chromatographed on silica gel (230–400 mesh) vacuum liquid chromatography (VLC) in different gradients of hexane/acetone/MeOH to 20% MeOH. From the six main fractions, three of them showed the new structures, corniculatolide B (**9**, 3 mg), isocorniculatolide B (**10**, 2 mg), and corniculatolide C (**11**, 5 mg), among some other known constituents. Different ^1^H and ^13^C NMR and HRESIMS techniques were employed to elucidate the chemical structures of the isolated compounds [19].

### 2 Synthesis

#### 2.1 Biosynthetic pathway

**Scheme 1:**
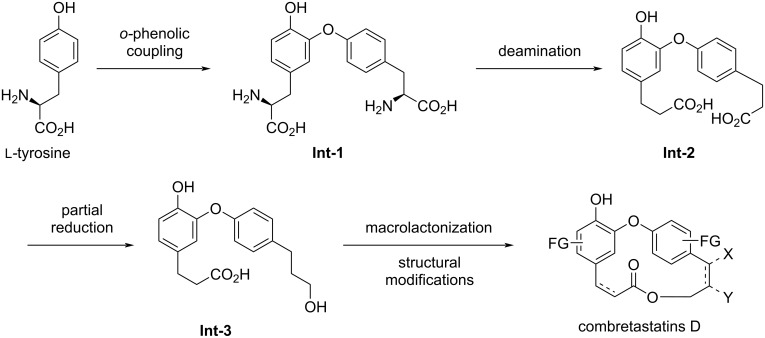
Biosynthetic pathway proposed by Pettit and co-workers.

In the literature, there are two possible biosynthetic pathways for the formation of these compounds. The first one was proposed by Pettit and co-workers [16–17] based on tyrosine as the starting material. An *o*-phenolic coupling between two units of tyrosine furnishes the intermediate **Int-1**, which by deamination, selective reduction of one of the carboxylate groups, macrolactonization, and subsequent structural modifications would lead to the aforementioned combretastatins D (Scheme 1).

**Scheme 2:**
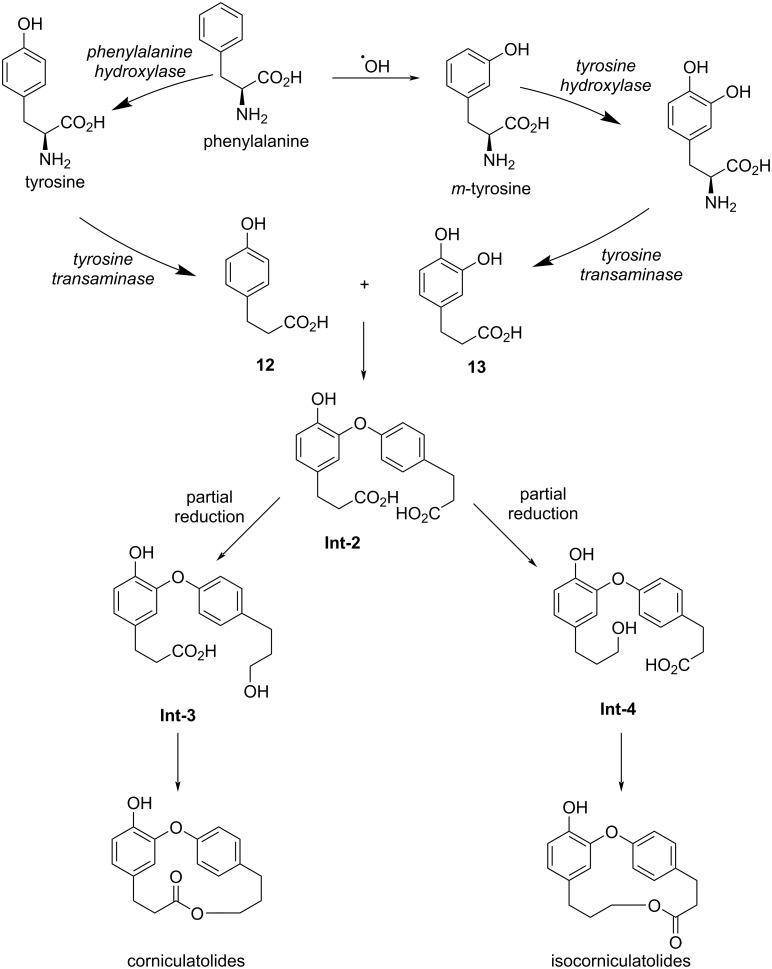
Biosynthetic pathway towards corniculatolides or isocorniculatolides proposed by Ponnapalli and co-workers.

The second pathway was proposed by Ponnapalli and co-workers [14] and was initially based on the conversion of phenylalanine into tyrosine by phenylalanine hydroxylase and *m-*tyrosine via radical hydroxylation (Scheme 2). Subsequent deamination of tyrosine, with concomitant hydroxylation/deamination of *m*-tyrosine would give compounds **12** and **13**. A coupling reaction would give the corresponding diaryl ether **Int-2**, in a similar way to that suggested by Pettit, which could be selectively reduced to afford the corresponding *seco-*acid (intermediates **Int-3** and **Int-4**). Subsequent macrolactonization would give the corniculatolides or isocorniculatolides.

#### 2.2 Retrosynthetic analysis

**Scheme 3:**
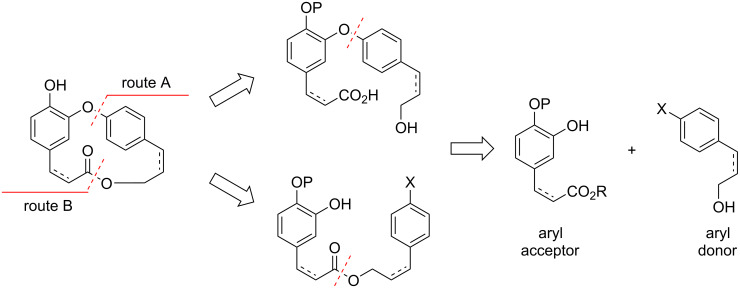
Retrosynthetic approaches.

Due to the aforementioned biological activities and the low availability from natural sources to provide sufficient material for additional investigations, the combretastatin D series and their isomeric macrolides have become an attractive target for synthesis. In general, the synthesis of these macrocyclic compounds can be accomplished by using two distinct disconnections (Scheme 3): one concerns the formation of the macrocycle through macrolactonization reaction from the former seco-acid formed from the Ar–O–Ar coupling from the aryl donor/acceptor (route A), while the other corresponds to the intramolecular Ar–O–Ar coupling from the former ester (route B).

Both synthetic routes have their advantages and disadvantages. The formation of the Ar–O–Ar bond can be accomplished using different methodologies [20] such as S_N_Ar [21], Ullmann [22], or Cham–Lam reactions [23–26]. However, it has been described that a Mitsunobu reaction of the *seco-*acid was particularly prone to an S_N_1 reaction, once the activated allylic alcohol yields an oxyphosphonium ion intermediate due to the conjugation to electron-rich aromatic ring, requiring some alternative experimental strategies to achieve the target molecule, as will be discussed further in this review.

#### 2.3 Synthesis of combretastatins D series

**Scheme 4:**
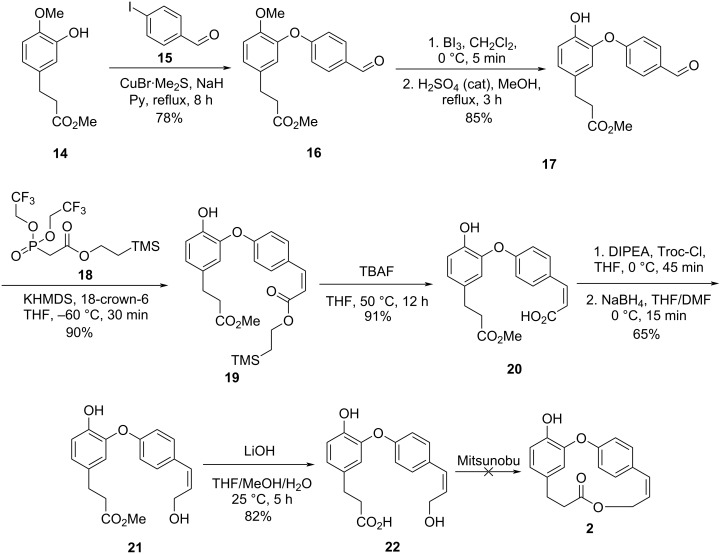
Attempt of total synthesis of **2** by Boger and co-workers employing the Mitsunobu approach [27].

Boger and co-workers were the first to report the total synthesis of compound **2** using both routes A and B to obtain the desired macrolide [27]. Initially, the authors employed an Ullmann-type condensation [28] between ester **14** and 4-iodobenzaldehyde (**15**) to give the corresponding diaryl ether **16** in 78% yield. The subsequent demethylation reaction using boron triiodide also promoted the hydrolysis of the ester, and thus a re-esterification step was necessary to give compound **17** in 85% yield after the two steps. Subsequent reaction of the aldehyde **17** following a modified Still–Gennari protocol [29] employing the phosphonate **18** gave the alkene **19** in 90% yield and high selectivity (*cis*/*trans* = 25:1). Removal of the silane group with TBAF furnished the carboxylic acid **20**, which underwent protection with Troc-Cl and selective reduction in the presence of sodium borohydride to form the alcohol **21**. After ester hydrolysis the desired *seco-*acid **22** was obtained in 82% yield. However, several attempts to achieve the macrolactonization of **22** using PPh_3_ and DEAD under different conditions [30] did not lead to the desired macrolide **2**, but only the formation of the diolide was observed (Scheme 4).

**Scheme 5:**
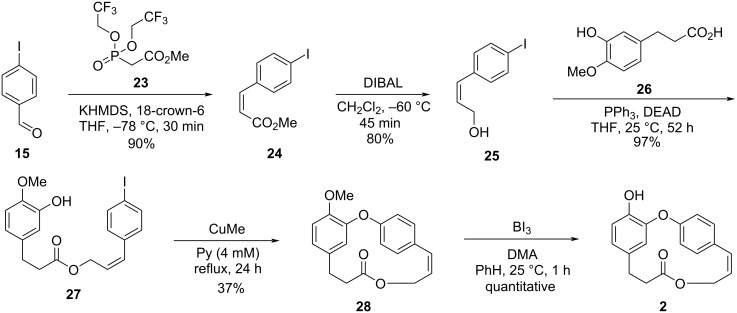
Total synthesis of combretastatin D-2 (**2**) reported by Boger and co-workers employing an intramolecular Ullmann reaction [27].

Once the first synthetic pathway did not furnish the desired compound, the authors carried out the formation of the macrocycle using an intramolecular Ullmann-type coupling reaction. Thus, the olefination reaction of aldehyde **15** with phosphonate **23**, followed by the reduction of the obtained ester **24** using DIBAL led to the alcohol **25**. The latter was submitted to the reaction with carboxylic acid **26** under Mitsunobu conditions [30], giving the corresponding ester **27** in 97% yield. Subsequent intramolecular Ullmann-type reaction using CuMe under high dilution conditions [31] gave macrolide **28** in low yield. Finally, demethylation using boron triiodide [32] led to the formation of combretastatin D-2 (**2**, Scheme 5).

Using this strategy, Boger succeeded to synthesize compound **2** in an overall yield of 25% after 5 steps, bypassing the macrolactonization problem evidenced in the previously envisaged route.

**Scheme 6:**
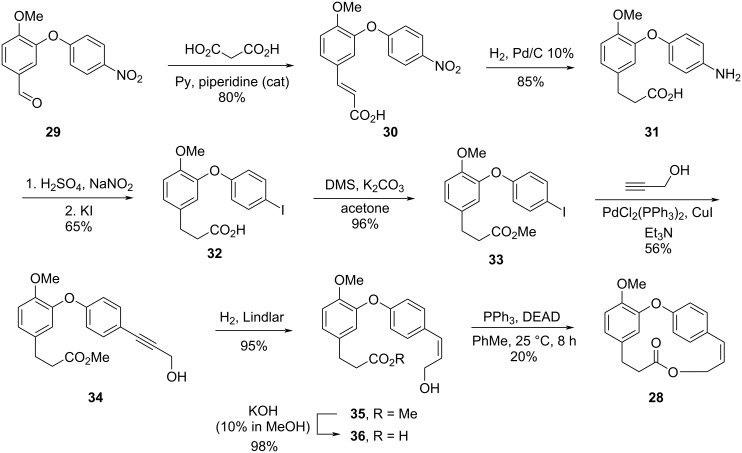
Formal synthesis of combretastatin D-2 (**2**) by Deshpande and co-workers using the Mitsunobu conditions in high dilution conditions [33].

Intrigued by the problem encountered by Boger, Deshpande decided to investigate different reaction conditions for the formation of the macrocycle using the Mitsunobu reaction [33]. Thus, Knoevenagel condensation using the diaryl ether **29** and malonic acid gave the corresponding α,β-unsaturated compound **30**, which was submitted to a concomitant hydrogenation of the double bond and the nitro group to give compound **31**. Sequential diazotization/halogenation and esterification reactions gave the ester **33** which was submitted to a Sonogashira coupling reaction with propargyl alcohol to give the advanced intermediate **34** [34]. Partial hydrogenation of the triple bond in **34** using Lindlar’s catalyst led to the *cis*-allylic alcohol **35** and subsequent ester hydrolysis led to the formation of *seco*-acid **36**. Macrolactonization attempts conducted under high dilution, including the Mitsunobu conditions [35] gave only the cyclic diolide. However, the use of higher dilution conditions and the dropwise addition of *seco*-acid **36** to a solution of DEAD (7.7 equiv) and triphenylphosphine (7.5 equiv) in PhMe led to the corresponding macrolide **28** in 20% yield. Since the demethylation step of **28** was known [27], the authors described a formal synthesis of combretastatin D-2 (**2**, Scheme 6).

Despite the low yield, the authors managed to bypass the dimerization reaction previously reported by Boger, and the formal synthesis of **2** was achieved after 8 steps with an overall yield of 4.5%.

Rychnovsky and Hwang hypothesized that the low yields from the Mitsunobu reaction in the previous synthesis of compound **2** were linked to the instability of the allylic oxyphosphonium ion formed with intermediates **22** and **36** (Scheme 4 and Scheme 6) and possibly an alkyl oxyphosphonium ion should be more stable [36]. Therefore, the authors proposed a synthetic sequence where the double bond was introduced only after the macrolactonization step.

**Scheme 7:**
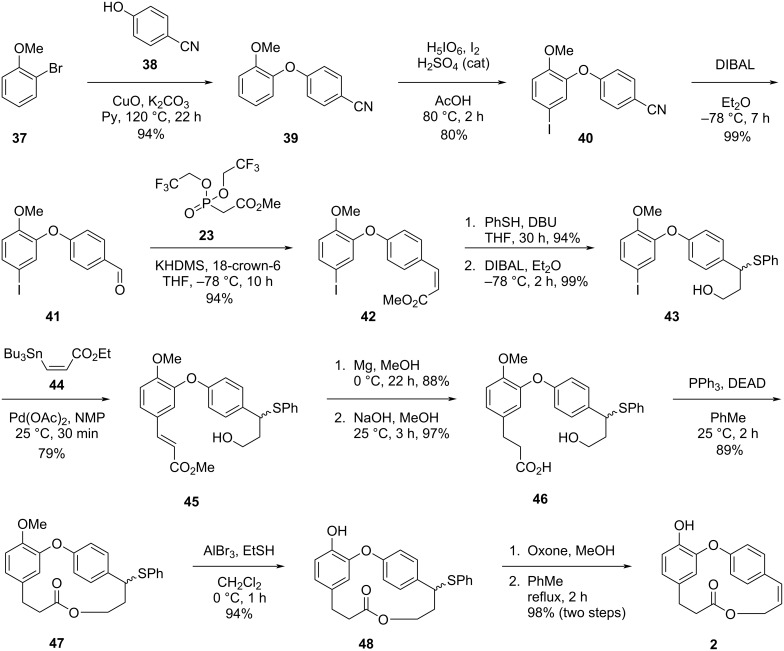
Total synthesis of combretastatin D-2 (**2**) by Rychnovsky and Hwang [36].

The synthetic route was initiated by an Ullmann-type coupling [37] between halide **37** and phenol **38** leading to the formation of diaryl ether **39**, which was subjected to a regioselective iodination reaction to give compound **40**. Conversion of the nitrile in compound **40** into the corresponding aldehyde **41** followed by *Z*-selective Still–Gennari olefination gave the *cis* α,β-unsaturated ester **42**. Conversion of the installed alkene to the corresponding thioether followed by the reduction of the ester moiety using DIBAL gave the compound **43**, which was subjected to a Stille coupling reaction [38] to yield compound **45**. Hydrogenation reaction in the presence of metallic Mg [39] followed by an ester hydrolysis led to the formation of *seco*-acid **46**. By using Mitsunobu conditions at high dilution and slow addition of reagents, the authors were able to synthesize the macrolide **47** in excellent yield, thus confirming their initial hypothesis regarding the stability of oxyphosphonium ions. Further demethylation [40] and oxidation of the thioether followed by thermal elimination of the intermediate sulfoxide gave **2** in 98% yield after two steps (Scheme 7).

**Scheme 8:**
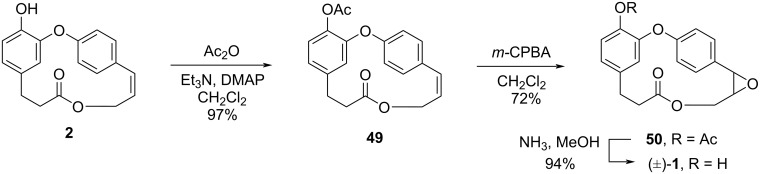
Divergent synthesis of (±)-**1** form combretastatin D-2 (**2**) by Rychnovsky and Hwang [36].

The authors also achieved the synthesis of (±)-**1** from combretastatin D-2 (**2**). Protection of the hydroxy group in compound **2** using acetic anhydride followed by epoxidation using *m*-CPBA gave protected epoxide **50**. Subsequent removal of the acetate group using ammonia led to racemic compound **1** (Scheme 8).

Rychnovsky and Hwang succeeded in the total syntheses of combretastatin D-2 (**2**) in a 36% overall yield after 13 steps and combretastatin D-1 (**1**) in 23% overall yield after 16 steps.

**Scheme 9:**
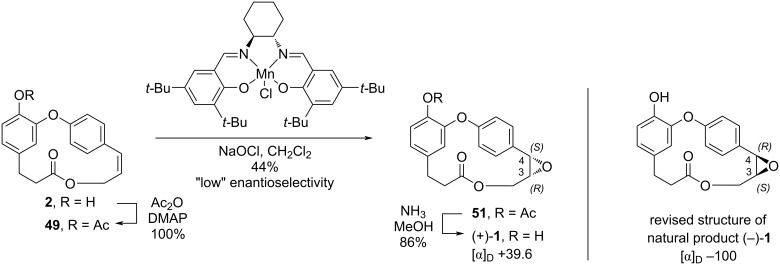
Enantioselective synthesis of **1** by Rychnovsky and Hwang employing Jacobsen catalyst [41].

Later, the same authors performed the enantioselective synthesis of **1** in an attempt to review its absolute configuration [41]. Thus, acetylation of compound **2** followed by the use of Jacobsen’s catalyst [42] to perform the epoxidation of the double bond gave the corresponding epoxide **51** in low enantioselectivity and only 44% yield. The subsequent deprotection reaction led to compound **1** in 86% yield (Scheme 9).

Besides the low enantioselectivity, the authors observed that the optical rotation value of the synthesized compound ([α]_D_ +36.9, *c* 0.55, CHCl_3_) was different from the value reported for the natural product ([α]_D_ −100, *c* 0.015, CHCl_3_) [16] and hypothesized that the configuration of the natural compound would be 3*S*,4*R,* different from that reported in the Pettit previous work.

Couladouros and co-workers based the synthetic design of combretastatin D on the use of computational calculations in order to find the intermediates with the lowest torsional energy for the cyclization step [43–45]. The authors came to the conclusion that both the formation of the double bond in compound **2** and the formation of the epoxide in compound **1** would only be favorable after the macrolactonization step.

**Scheme 10:**
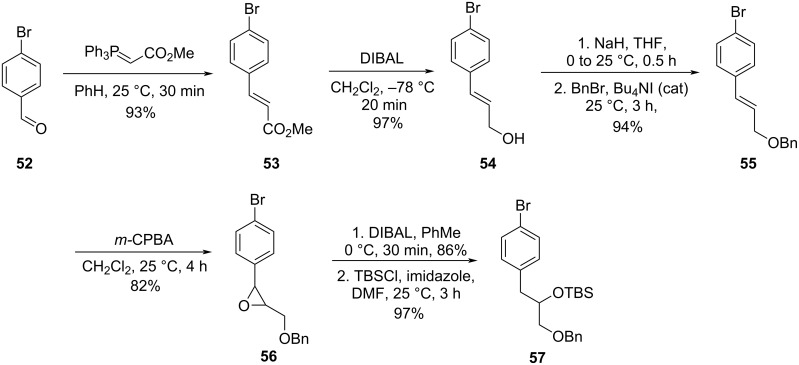
Synthesis of fragment **57** by Couladouros and co-workers [43,45].

The authors used a convergent route for the formal synthesis of **2**. Reaction of 4-bromobenzaldehyde (**52**) with a commercially available stabilized Wittig reagent led to the formation of the corresponding ester **53**. Further reduction of the carbonyl group followed by protection of the obtained alcohol with benzyl bromide provided compound **55**, which was subjected to an epoxidation using *m*-CPBA followed by ring opening using DIBAL [46]. The obtained alcohol was then protected with TBSCl to give fragment **57** (Scheme 10).

**Scheme 11:**
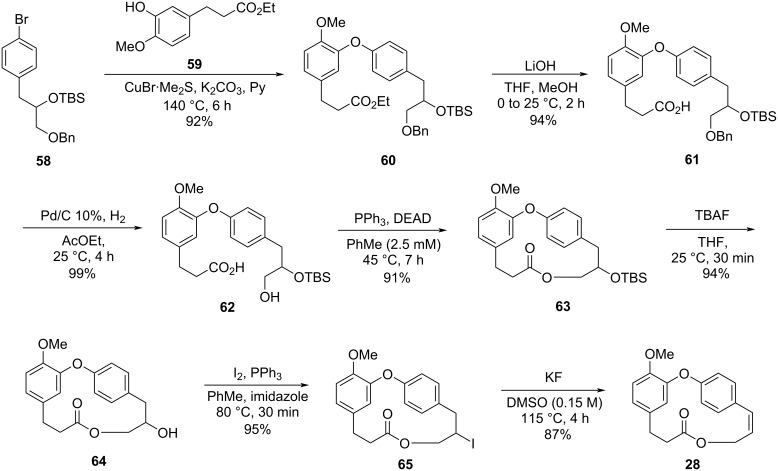
Formal synthesis of compound **2** by Couladouros and co-workers [43,45].

Using similar conditions to Boger´s protocol, compound **58** was then subjected to an Ullmann coupling reaction in the presence of ester **59** to yield the corresponding diaryl ether **60**. The hydrolysis of the ester followed by the removal of the benzyl group led to the corresponding *seco*-acid **62**. The obtained compound showed high stability when subjected to Mitsunobu conditions with the slow addition of the *seco*-acid into the DEAD/PPh_3_ reaction mixture over a 7 h period. The desired macrolide **63** was obtained in 91% yield, without formation of the diolide being observed. Subsequent removal of the TBS group gave the corresponding alcohol **64**. The formation of the double bond from alcohol **64** proved to be problematic, thus, replacement of the hydroxy group by iodine [47] followed by dehydrohalogenation using an excess of KF afforded methyl combretastatin D-2 (**28**) in 87% yield after two steps (Scheme 11).

**Scheme 12:**
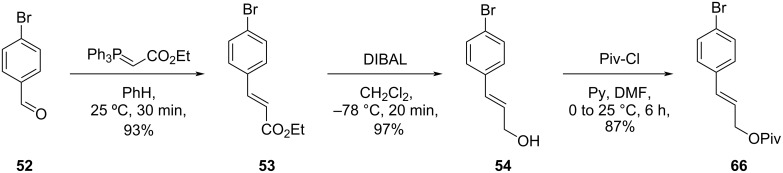
Synthesis of fragment **66** by Couladouros and co-workers [44–45].

The authors also described a convergent route by which it was possible to reach both, compound **2** and **1**. Initially, the authors prepared the protected alcohol **66** from aldehyde **52** in a linear sequence (Scheme 12).

**Scheme 13:**
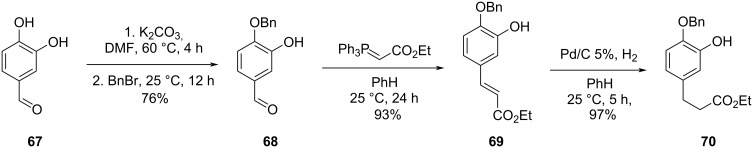
Synthesis of fragment **70** by Couladouros and co-workers [44–45].

In parallel, the monobenzylation of 3,4-dihydroxybenzaldehyde (**67**), followed by chain elongation using the Wittig reaction furnished the α,β-unsaturated ester **69**. The subsequent catalytic hydrogenation led to the desired phenol **70** (Scheme 13) [44–45].

**Scheme 14:**
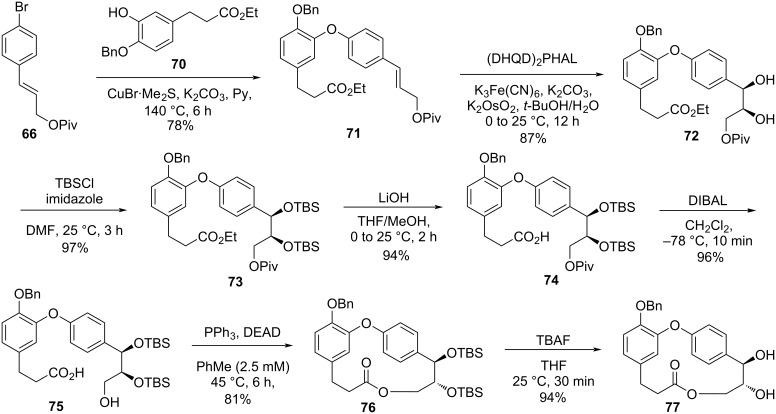
Synthesis of fragment **77** by Couladouros and co-workers [44–45].

An Ullmann coupling reaction using compounds **66** and **70** gave the corresponding diaryl ether **71**, which was submitted to an asymmetric dihydroxylation reaction using (DHQD)_2_PHAL to yield diol **72**. The 3*R*,4*S* configuration of compound **72** was expected based on Pettit’s work [16–17] and the optical purity of the obtained product was more than 95% by ^1^H NMR using [Eu(hfc)_3_] as a chiral shift reagent. Subsequent silylation followed by ester hydrolysis and removal of the pivaloyl group provided the *seco*-acid **75**. Employing the same Mitsunobu conditions previously described [35], the authors were able to obtain the macrolide **76** in 81% yield which was then subjected to deprotection to give compound **77** (Scheme 14).

**Scheme 15:**
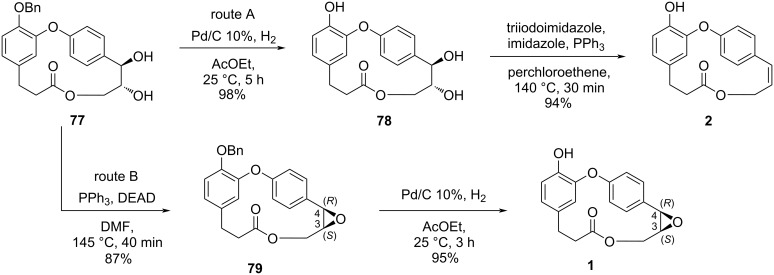
Synthesis of combretastatins **1** and **2** by Couladouros and co-workers [44–45].

The synthesis of **2** from compound **77** was achieved after hydrogenolysis of the benzyl ether. Further double bond formation in compound **78** employing triiodoimidazole and PPh_3_ led to **2** (route A, 32% overall yield from **52**). The synthesis of combretastatin D-1 (**1**) was achieved from the cyclodehydration of compound **77**, followed by the hydrogenolysis of the benzyl ether **79** (route B, overall yield of 29% from **52**) (Scheme 15).

Using this strategy, combretastatin D-1 (**1**) was obtained in an enantiomeric purity of 96%, making it possible to establish the absolute configuration of the epoxide. Further, X-ray crystallographic analysis corroborated with the results obtained by Rychnovsky and Hwang [41] that the correct configuration was 3*S*,4*R*, evidencing that the original attribution described by Pettit and co-workers [16–17] was inaccurate.

**Scheme 16:**
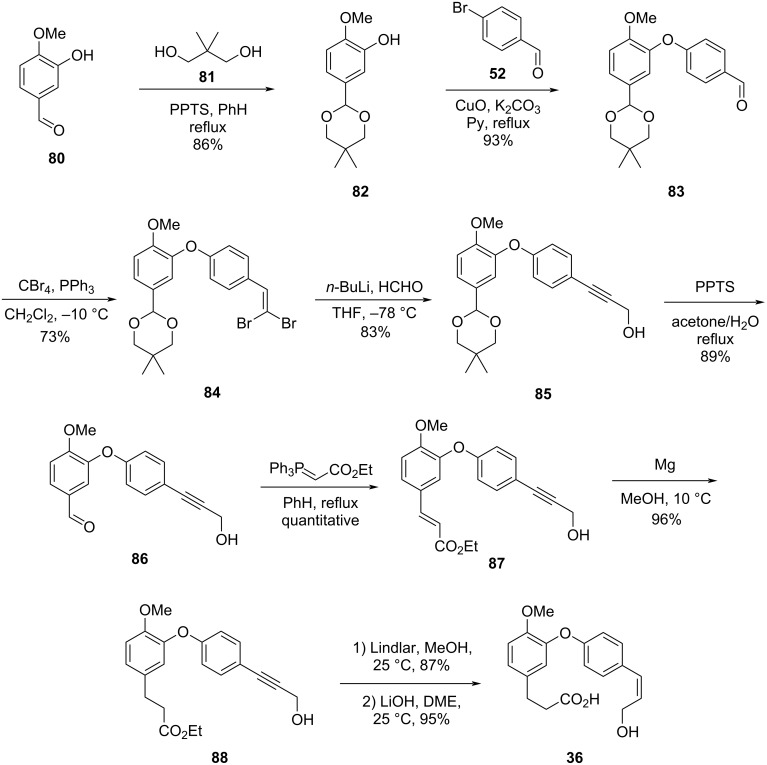
Formal synthesis of compound **2** by Gangakhedkar and co-workers [48].

Gangakhedkar elaborated a synthetic route where it was possible to formally synthesize compound **2** in 9 steps [48]. Conversion of the hydroxybenzaldehyde **80** into the corresponding acetal followed by Ullmann-type coupling with **52**, led to the formation of diaryl ether **83**. Subsequent Corey–Fuchs reaction [49] and in situ alkylation led to formation of the propargylic alcohol **85**. Deprotection of the aldehyde followed by chain elongation through the Wittig reaction led to the α,β-unsaturated ester **87**, which was subjected to a hydrogenation reaction in the presence of metallic magnesium, leading to the formation of alkyne **88**. The *cis-*alkene was selectively obtained using the Lindlar catalyst. Finally, hydrolysis of the ester led to the formation of *seco*-acid **36**. Using this approach, the authors were able to achieve the formal synthesis of **2** reaching a key intermediate in 34% overall yield after 9 steps (Scheme 16).

**Scheme 17:**
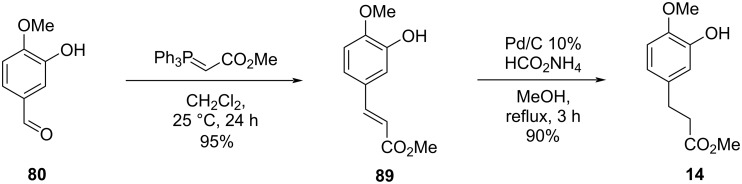
Synthesis of fragment **14** by Cousin and co-workers [50].

Cousin and co-workers [50] innovated by using the Chan–Lam coupling [23–25] for the diaryl ether formation and applying an intramolecular Wittig reaction to promote the macrocyclization in the formal synthesis of compound **2**. Initially, the authors synthesized the fragment **14** from the starting aldehyde **80** by using a Wittig reaction followed by hydrogenation using ammonium formate (Scheme 17).

**Scheme 18:**
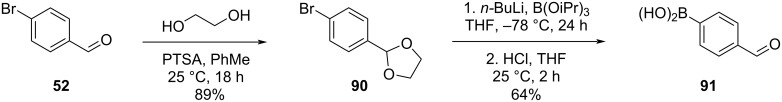
Synthesis of fragment **91** by Cousin and co-workers [50].

Concomitantly, 4-formylphenylboronic acid (**91**) was prepared from the borylation reaction of 4-bromobenzaldehyde (**52**, Scheme 18) [50].

**Scheme 19:**
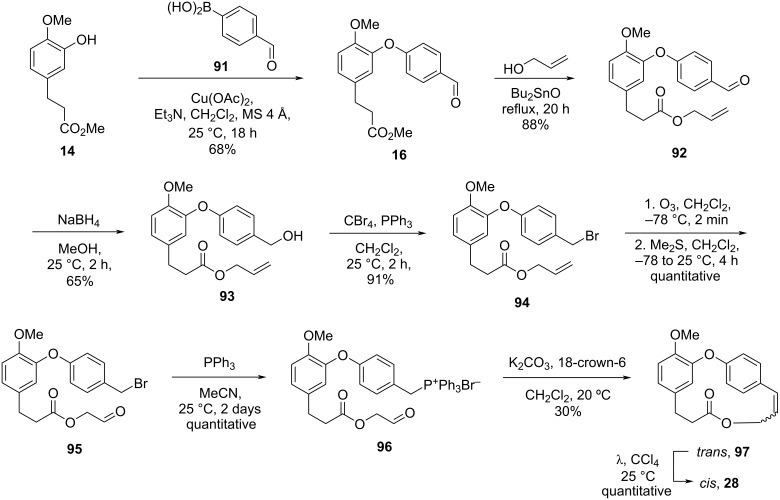
Formal synthesis of compound **2** by Cousin and co-workers [50].

The coupling reaction [23–25] between **14** and **91** gave the corresponding diaryl ether **16** in 68% yield. Subsequent transesterification reaction [51] using dibutyltin oxide and allylic alcohol led to the formation of compound **92**, which was reduced to the corresponding alcohol and then converted into the bromide **94**. Ozonolysis followed by reaction with triphenylphosphine gave the corresponding phosphonium salt **96**, which was subjected to different conditions for the intramolecular Wittig reaction. The best conditions found by the authors gave the desired macrolide in only 30% yield together with the *trans* isomer, which was further isomerized to the *cis*-alkene during purification by column chromatography and light, being the first time that the *trans* isomer was reported (Scheme 19).

**Scheme 20:**
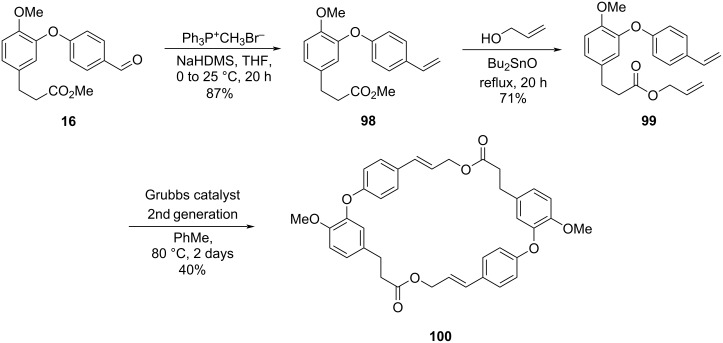
Synthesis of **2** diolide by Cousin and co-workers [50].

Another strategy employed by the authors consisted in the ring closure through a metathesis reaction using the Grubbs catalyst [52–53]. The required compound **99** was prepared by converting compound **16** into the styrene **98** via a Wittig reaction followed by a transesterification to yield the desired allylic ester. Several reaction conditions for the metathesis using the 1st generation Grubbs catalyst were attempted without success, but when 2nd generation catalyst was used, the dimerization product **100** was observed (Scheme 20).

Despite the yield for macrolide formation, Cousin proposed alternatives for the formal synthesis of **2**, employing a 10-step synthetic route with an overall yield of 9%.

**Scheme 21:**
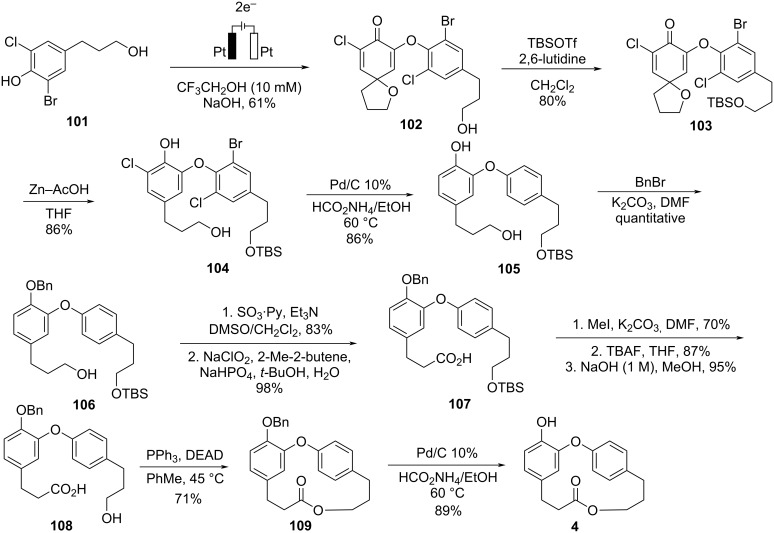
Synthesis of combretastatin D-4 (**4**) by Nishiyama and co-workers [54].

Nishiyama employed electrochemical techniques as a starting point to achieve the total synthesis of combretastatin D-4 (**4**) [54]. Different anodic oxidation conditions and phenolic substrates were tested aiming at the formation of a diaryl ether moiety. The best result was obtained when phenol **101** was subjected to anodic oxidation, leading to the formation of *spiro*-dimer **102** in 61% yield. Protection of the alcohol using TBSOTf followed by cyclic ether cleavage and re-aromatization gave compound **104**. Subsequent dehalogenation followed by protection with BnBr and oxidation led to the carboxylic acid **107**. Esterification of the carboxylic acid followed by the cleavage of the silyl ether using TBAF and hydrolysis led to the *seco*-acid **108**. Macrolactonization using the Mitsunobu conditions gave combretastatin D-4 (**4**) after cleavage of the benzyl ether using Pd/C and ammonium formate (Scheme 21).

With this synthetic route, the authors achieved the total synthesis of combretastatin D-4, after 12 reaction steps with an overall yield of 11%, highlighting the efficient formation of the diaryl ether **102** in 61% yield without the use of metallic catalysts, through dimerization of a single molecule.

**Scheme 22:**
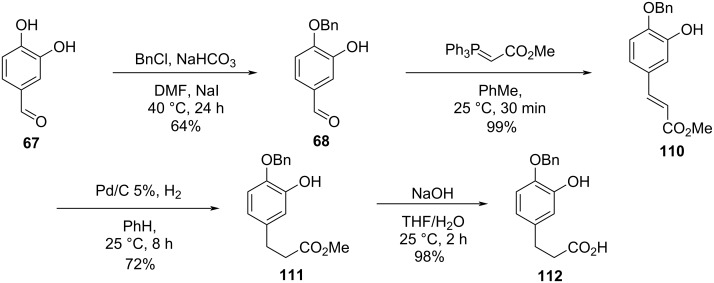
Synthesis of fragment **112** by Pettit and co-workers [55].

Pettit and co-workers investigated the influence of structural modifications on the biological activity of combretastatins D-2 (**2**) and D-4 (**4**). The authors also investigated the influence of solvents and functional groups in the total synthesis of the targeted compounds aiming to reach higher overall yields and fewer steps [55]. The monobenzylation [56] of aldehyde **67** followed by chain elongation using a Wittig reaction gave compound **68**, which was submitted to the hydrogenation of the double bond. The use of benzene as solvent in the hydrogenation step proved to be important for the selectivity of the reaction, where significant cleavage of the benzyl group resulted when ethanol was the solvent of choice. Subsequent ester hydrolysis gave compound **112** (Scheme 22) [55].

**Scheme 23:**
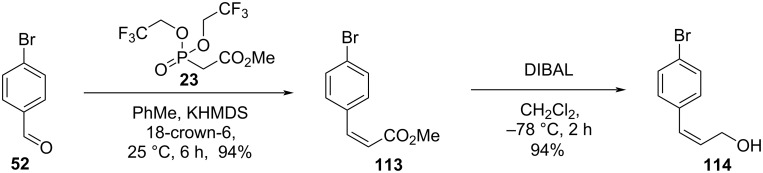
Synthesis of fragment **114** by Pettit and co-workers [55].

In parallel, a Still–Gennari olefination using aldehyde **52** lead to the *cis*-alkene **113**, which was reduced to the corresponding allylic alcohol **114** using DIBAL (Scheme 23) [55].

**Scheme 24:**
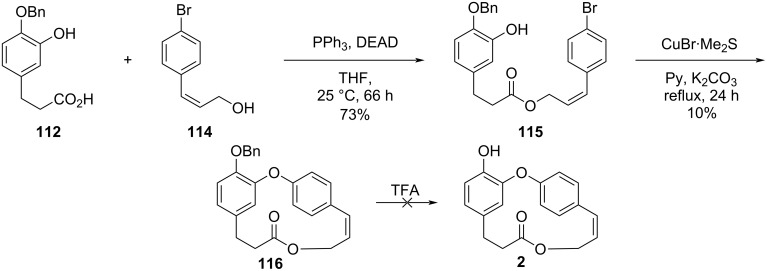
Attempt to the synthesis of compound **2** by Pettit and co-workers [55].

Further reaction of fragments **112** and **114** under Mitsunobu conditions gave the corresponding ester **115** in 73% yield. For the subsequent intramolecular Ullmann reaction varying equivalents of CuBr·Me_2_S complex and potassium carbonate or methylcopper in pyridine led to compound **116** in only 10% yield. The cleavage of the benzyl ether proved to be complicated, as TFA also opened the lactone at the ester group (Scheme 24) [55].

**Scheme 25:**
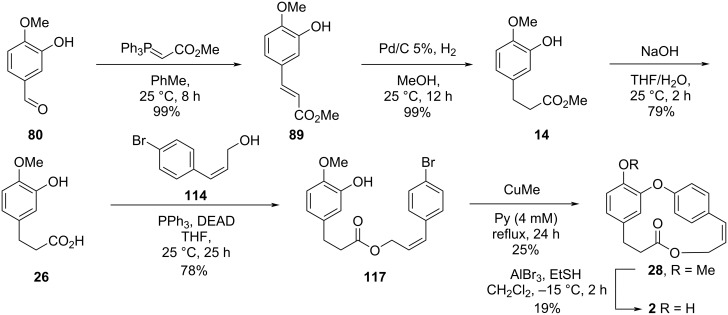
Synthesis of combretastatin-D2 (**2**) starting from isovanilin (**80**) by Pettit and co-workers [55].

In an attempt to circumvent these problems, the authors chose to use isovanillin (**80**) as starting compound in a similar synthetic sequence to synthesize the coupling substrate **26**. When the Mitsunobu conditions were applied for the reaction between **26** and the allylic alcohol **114**, the corresponding ester **117** was obtained in 78% yield, and the intramolecular Ullmann reaction using CuMe led to the formation of *O*-methylcombretastatin D-2 (**28**) in 25% yield. Further demethylation in the presence of aluminum tribromide and ethanethiol gave combretastatin D-2 (**2**) in 19% yield. Using this approach, Pettit obtained the desired compound in approximately 3% overall yield after 6 steps (Scheme 25) [55].

**Scheme 26:**
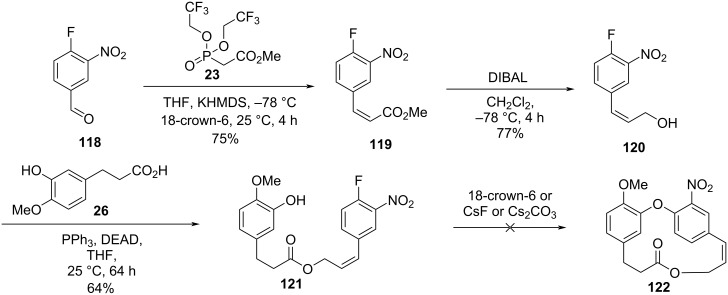
Attempted synthesis of combretastatin-D2 (**2**) derivatives through an S_N_Ar approach [55].

In order to obtain higher yields in the intramolecular cyclization step, the authors also investigated the use of a strategy based on an S_N_Ar reaction using an electron-deficient aryl halide. Thus, 4-fluoro-3-nitrobenzaldehyde (**118**) was subjected to the Still–Gennari reaction, to give the corresponding *cis*-olefin **119** which was reduced using DIBAL to lead to the allylic alcohol **120**. Subsequent Mitsunobu reaction between the alcohol **120** and carboxylic acid **26** gave the corresponding ester **121** in a 64% yield. However, the intramolecular cyclization step did not lead to the desired compound **122**, even when different types of bases and reaction conditions were used (Scheme 26).

**Scheme 27:**
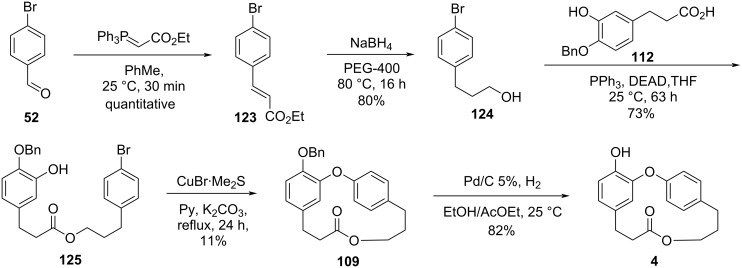
Synthesis of combretastatin D-4 (**4**) by Pettit and co-workers [55].

Finally, the authors synthesized combretastatin D-4 (**4**) starting from the Wittig reaction between aldehyde **52** and a commercially available Wittig reagent to give the corresponding α,β-unsaturated ester **123** in quantitative yield. Subsequent one-step reaction using sodium borohydride and polyethylene glycol gave directly alcohol **124**, which was subjected to the Mitsunobu reaction with the carboxylic acid **112**. The obtained ester **125** was then used in an intramolecular Ullmann reaction to yield the benzylated combretastatin D-4, **109** in only 11% yield. Further cleavage of the benzyl ether gave combretastatin D-4 (**4**) in 5% overall yield after 5 steps (Scheme 27).

Pettit contributed significantly to the synthesis of members of the combretastatin D series, verifying the viability of different reaction routes, the influence of different functional groups to obtain the desired compounds and especially with regard to the structure–activity relationship of these molecules against different types of cancer cells (see Section 3).

**Scheme 28:**
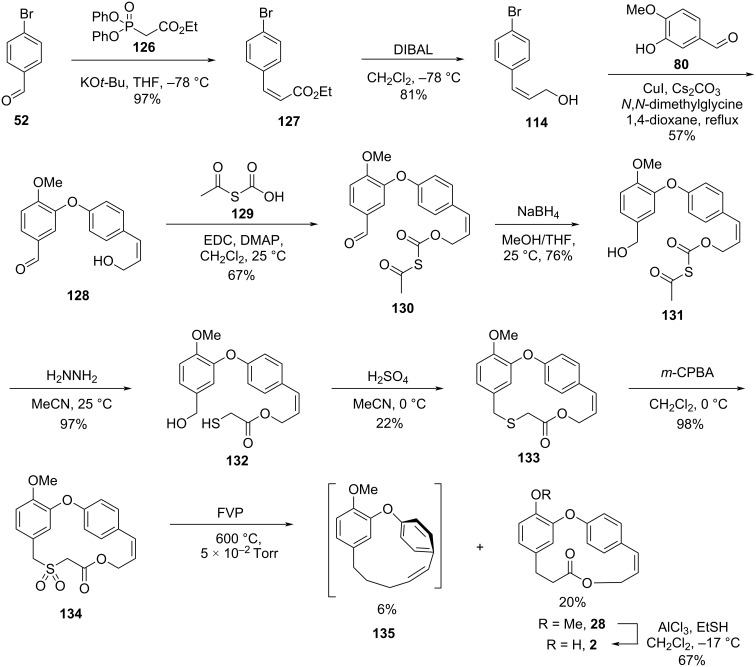
Synthesis of combretastatin D-2 (**2**) by Harras and co-workers [57].

Harras and co-workers [57] achieved the total synthesis of combretastatins D-2 (**2**) and D-4 (**4**) and the formal synthesis of combretastatin D-1 (**1**) using the flash vacuum pyrolysis (FVP) technique, which consisted on the contraction of 16-membered sulfone derivatives, by extrusion of sulfur dioxide [58]. The synthesis of the required sulfone was initiated by a Horner–Wadsworth–Emmons reaction between the aldehyde **52** and the phosphonate **126** leading to the *cis*-ester **127** in high yield. Reduction of the ester using DIBAL gave the allylic alcohol **114** which was submitted to an Ullmann coupling [59] with isovanillin (**80**) to give the corresponding diaryl ether **128**. Further esterification [60] with *S*-acetylthioacetic acid (**129**) followed by the reduction [61] of the aldehyde gave the corresponding benzyl alcohol **131** in 76% yield. Deacetylation in the presence of hydrazine [62] followed by intramolecular thioetherification led to the macrocycle **133**. Finally, the oxidation of the obtained thioether to the corresponding sulfone **134** using *m*-CPBA [63] followed by contraction of the macrocyclic ring by extrusion of SO_2_ using FVP gave compound **28** together with macrocycle **135**, obtained from the simultaneous extrusion of SO_2_ and CO_2_. Cleavage of the methyl ether in **28** gave the desired product **2** in 0.6% overall yield after 10 steps (Scheme 28) [57].

**Scheme 29:**
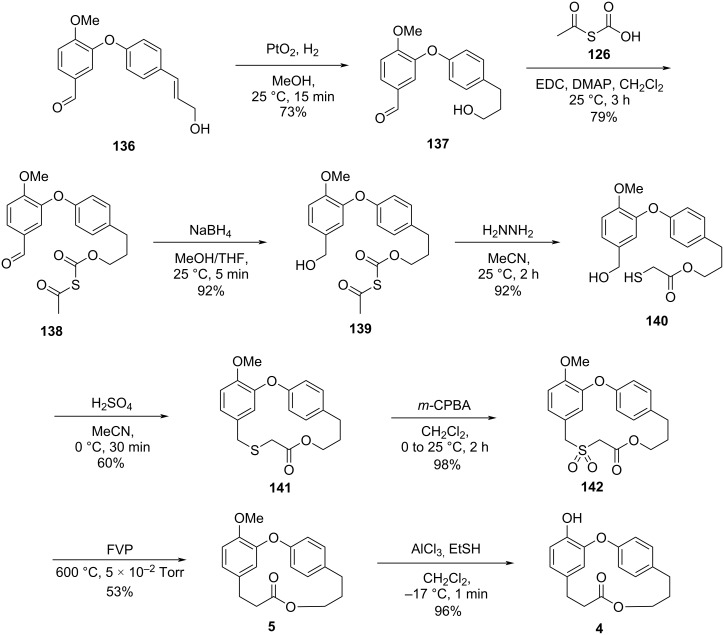
Synthesis of combretastatin D-4 (**4**) by Harras and co-workers [57].

Using the same synthetic approach, the authors achieved the total synthesis of combretastatin D-4 (**4**) starting with the hydrogenation of diaryl ether **136** (Scheme 29) [57].

**Scheme 30:**
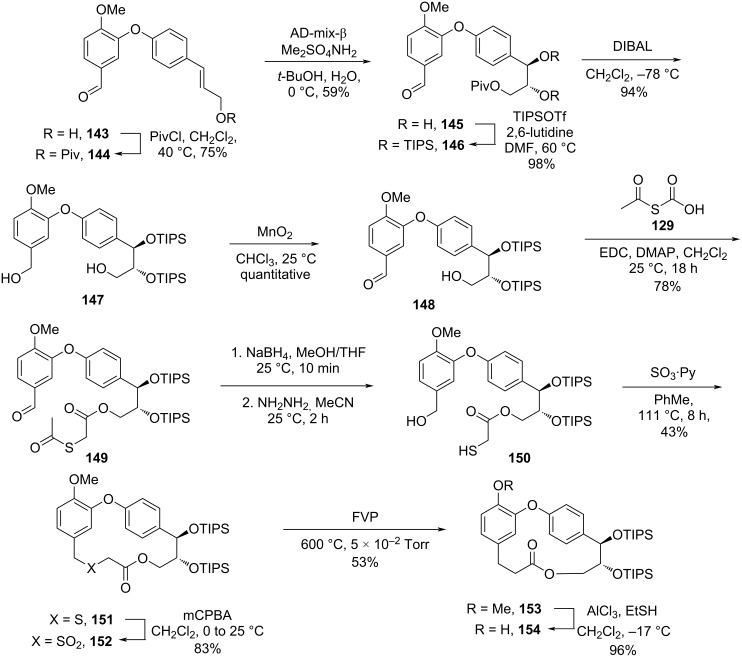
Formal synthesis of combretastatin D-1 (**1**) by Harras and co-workers [57].

The authors also achieved the formal synthesis of combretastatin D-1 (**1**). Starting from the protection of alcohol **143** with pivaloyl chloride [64] and subsequent dihydroxylation of the double bond in **144** according to the Sharpless protocol using AD-mix-β [65], furnished the required *syn*-diol **145** in 59% yield and >99% ee. The hydroxy groups were protected [66] as TIPS ethers **146** and treatment with DIBAL-H led to both, cleavage of the Piv group and reduction of the aldehyde yielding the diol **147**. Selective oxidation [67] of the benzyl alcohol with MnO_2_ gave the compound **148**, which was esterified with *S*-acetylthioacetic acid (**129**) and reduced to the benzyl alcohol **150**. Deacetylation followed by macrocyclization using SO_3_·pyridine [68] gave the corresponding thioether **151**, which was oxidized to the cyclic sulfone **152** using *m*-CPBA. Extrusion of SO_2_ by FVP followed by demethylation of the formed macrolide furnished the compound **154** which can be converted in combretastatin D-1 (**1**) by known methodologies [43] (Scheme 30) [57].

Using this synthetic approach, the authors were capable to achieve the total synthesis of combretastatins **2**, **4** and the formal synthesis of **1** in 9–10 steps in average global yields around one percent.

#### 2.4 Synthesis of corniculatolides and isocorniculatolides

**Scheme 31:**
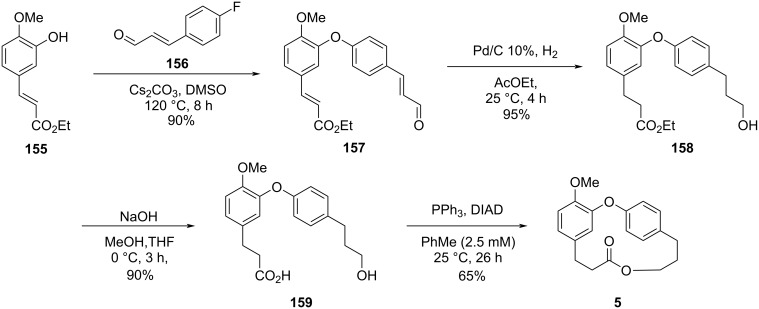
Synthesis of 11-*O*-methylcorniculatolide A (**5**) by Raut and co-workers [69].

Raut developed a synthetic route for the preparation of isomeric macrolides of combretastatin D congeners called 11-*O*-methylcorniculatolide A (**5**), isocorniculatolide A (**7**), and 11-*O*-methylisocorniculatolide A (**8**), where the key steps comprised an S_N_Ar reaction for the diaryl ether formation and a Mitsunobu reaction for the macrolide formation [69]. Thus, the S_N_Ar reaction between the ester **155** and the aldehyde **156** led to the formation of diaryl ether **157** which was subjected to a hydrogenation reaction followed by hydrolysis of the ester group to yield the corresponding *seco*-acid **159**. Subsequent Mitsunobu reaction led to 11-OMe-corniculatolide A (**5**) in a 50% overall yield after 4 steps (Scheme 31) [69].

The authors did not isolate dimers or oligomers using this strategy and attributed this fact to the slow addition of the acyclic precursor **159** to a solution of Ph_3_P and DIAD (2.5 mM in PhMe).

**Scheme 32:**
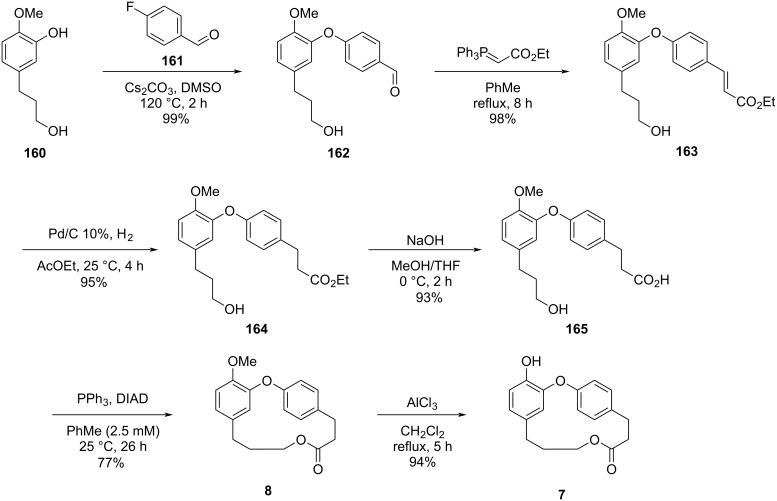
Synthesis of isocorniculatolide A (**7**) and *O*-methylated isocorniculatolide A **8** by Raut and co-workers [69].

Using a similar strategy, the authors carried out the reaction with compound **160** [70] and *p*-fluorobenzaldehyde (**161**) to obtain diaryl ether **162**. Chain elongation using a commercially available stabilized Wittig reagent followed by hydrogenation of the double bond provided compound **164**, which was hydrolyzed to the corresponding *seco*-acid **165**. Employing again the Mitsunobu conditions, 11-OMe-isocorniculatolide A (**8**) was obtained in 85% overall yield after 5 steps. Subsequent cleavage of the methyl ether using aluminum chloride led to isocorniculatolide A (**7**) in 94% yield after 6 reaction steps and an overall yield of 62% (Scheme 32) [69].

**Scheme 33:**
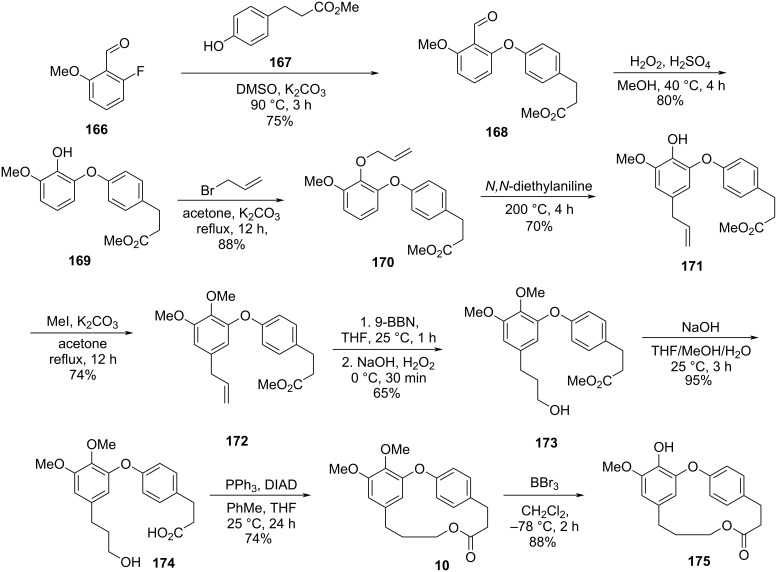
Synthesis of isocorniculatolide B (**10**) and hydroxyisocorniculatolide B **175** by Kim and co-workers [71].

Through a divergent synthetic route, employing as main steps the formation of the diaryl ether through S_N_Ar-type reactions and the macrolide formation using the Mitsunobu reaction, Kim and co-workers [71] synthesized isocorniculatolide B and corniculatolides B and C for further evaluation of their anti-inflammatory activity (see Section 3). Reaction of compounds **166** and **167** gave the corresponding diaryl ether **168**, which was converted to phenol **169** using a Baeyer–Villiger oxidation reaction followed by hydrolysis. Subsequent phenol allylation reaction followed by Claisen rearrangement led to the formation of compound **171**, which was methylated and subjected to a hydroboration reaction using 9-BBN. Further oxidation gave compound **173** in 65% yield. Hydrolysis of **173** gave the corresponding *seco*-acid **174**, which was subjected to a Mitsunobu reaction, to give isocorniculatolide B (**10**) in 12% overall yield after 8 steps. Selective demethylation led to hydroxyisocorniculatolide B, **175** in 11% overall yield after 9 reaction steps (Scheme 33) [71].

**Scheme 34:**
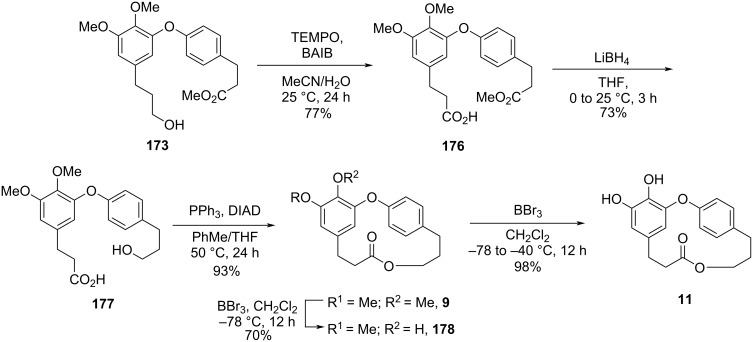
Synthesis of compound **9**, **178**, and **11** by Kim and co-workers [71].

The intermediate **173** was also submitted to an oxidation using BAIB and TEMPO [72], followed by reduction of the ester using LiBH_4_ to provide the *seco*-acid **177**. Further macrolactonization using Mitsunobu conditions led to the methylated compound **9** in 9% overall yield after 9 steps. Compound **9** is the precursor of both, hydroxycorniculatolide B **178** (6.5% overall yield after 10 steps) and corniculatolide C (**11**, 8% overall yield after 10 steps) (Scheme 34) [71].

Through this synthetic sequence, the authors were able to obtain the target compounds in global yields ranging from 6% to 13% in up to 10 steps.

### 3 Biological activities

Before the isolation of combretastatin D and its analogues, there were reports of the use of plant extracts containing these compounds in folk medicine for the treatment of different types of diseases, such as inflammatory processes, viral infections, metabolic disorders and some types of cancer [13–19].

The first biological studies of the combretastatin D series exploited their anticancer activity, since Pettit and co-workers, in a program from U.S. National Cancer Institute for the discovery of new anticancer agents, initially showed that combretastatins D-1 (**1**) and D-2 (**2**) isolated from the bark of the *Combretum caffrum* tree inhibited the growth of the murine lymphocytic leukemia cell line P388 with a median effective dose values (ED_50_) of 3.3 and 5.2 μg·mL^−1^ (10.56 and 17.55 μM), respectively [16–17].

Vongvanich and co-workers performed a cytotoxicity assay of combretastatins D-3 (**3**) and D-4 (**4**) against human breast cancer cells (BC-1), human epidermoid carcinoma of the mouth (KB), a small-cell lung cancer cell line (NCI-H187) and Vero cell lines using a colorimetric method and employing ellipticine as reference drug. Combretastatin D-3 (**3**) showed low activity against the small-cell lung cancer cell line with a half-maximal inhibitory concentration (IC_50_) of 13.0 μg·mL^−1^ (40 μM), but was inactive to the other cell lines. Unfortunately, combretastatin D-4 (**4**) was inactive to all cancer cell lines tested (Table 1) [18].

**Table 1:** Results from cytotoxicity assays of combretastatin D-3 (**3**) and D-4 (**4**) against cancer cell lines [18].

Compound	IC_50_ μg·mL^−1^ (molar concentration)^a^			

	KB	BC-1	NCI-H187	Vero cells

**3**	>20 (>60 μM)	>20 (>60 μM)	13.0 ± 0.2 (40 μM)	>50 (>150 μM)
**4**	>20 (>65 μM)	>20 (>65 μM)	>20 (>65 μM)	>50 (>160 μM)
ellipticine	0.2–0.3 (≈0.8 μM)			

^a^Approximated values of molar concentration after conversion.

Later, Nishiyama and co-workers evaluated combretastatin D-4 (**4**) against proliferation of human HT-29 colon carcinoma cells and observed a value of IC_50_ of 18.4 µg·mL^−1^ (61.8 μM) [54].

*O*-Methylcombretastatin D-2, **28** was also evaluated for its antiproliferative activity against MCF-7 human breast carcinoma, RKO human colon carcinoma, and CRL 1730 human umbilical endothelial cells. It exhibited activity in all cell lines with IC_50_ values around 5–10 mM [50].

Aiming to investigate the structure–activity relationship (SAR) of combretastatin D-2, Couladouros and co-workers [73] studied the effect of structural modifications in compound **28** on tubulin polymerization at different concentrations using the filtration-colorimetric method. Tubulin polymerization results in the formation of microtubules, which are important structures in the constitution of eukaryotic cells [74]. All tested compounds interfered with the polymerization of tubulin, and when compared to colchicine, classified as a destabilizing agent which prevents the microtubule assembly [55], the tested derivatives of compound **2** favor to various degrees the formation of microtubules, suggesting that the mechanism of this class of compound interacts with tubulin in a way to allow the microtubule assembly. The authors also observed that the introduction of higher polarity groups at the position of the double bond of **28** leads to compounds of increasing activity, being the more polar hydroxy-substituted derivatives the most active (Table 2) [73].

**Table 2:** Effect of combretastatin D derivatives on tubulin polymerization (%) [73].

	Concentration (mM)			

	0.25	0.5	1.0	2.0

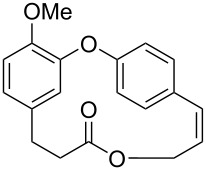 **28**	16	19	27	35
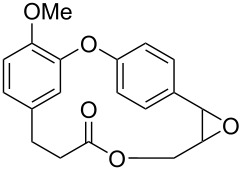 **179**	0	20	27	39
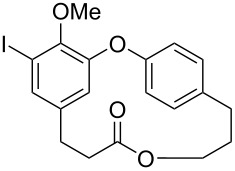 **180**	5	28	35	54
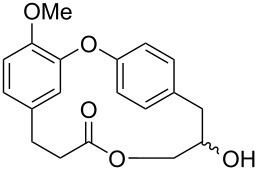 **181**	27	47	62	76
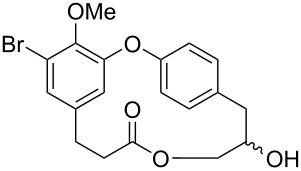 **182**	3	21	39	67
colchicine	4	3	4	0

**Scheme 35:**
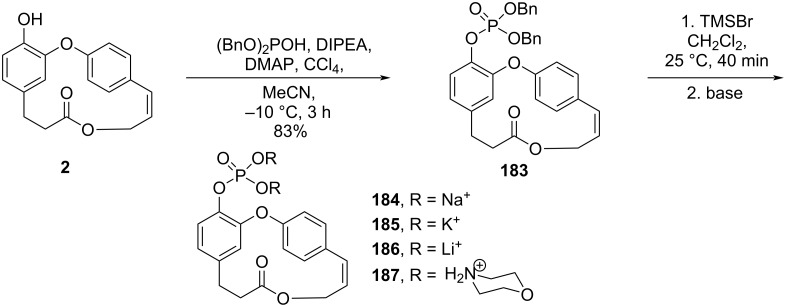
Synthesis of combretastatin D-2 prodrug salts [55].

One of the major obstacles in the development of highly potent drugs is the water solubility. Pettit and co-workers [55] conducted an extensive study in an attempt to improve the water solubility of combretastatin D-2 (**2**) by converting it into a series of phosphate salts and other prodrugs. Thus, phosphorylation of combretastatin D-2 (**2**) using dibenzyl phosphite gave derivative **183**. Further cleavage of the benzyl groups using TMSBr followed by the reaction of the obtained phosphoric acid derivative with a series of bases led to the formation of different phosphate salts **184**–**187** (Scheme 35) [55].

The authors observed that the prodrug salts had substantially improved solubility in water, an important feature for transport to metastatic cancer (Table 3) [75].

**Table 3:** Solubility comparison of combretastatin D-2 (**2**) and prodrugs in water at 25 °C [55].

Compound	Solubility (mg·mL^−1^)

**2**	0.5
**184**	>70
**185**	>50
**186**	20
**187**	5

**Figure 2:**
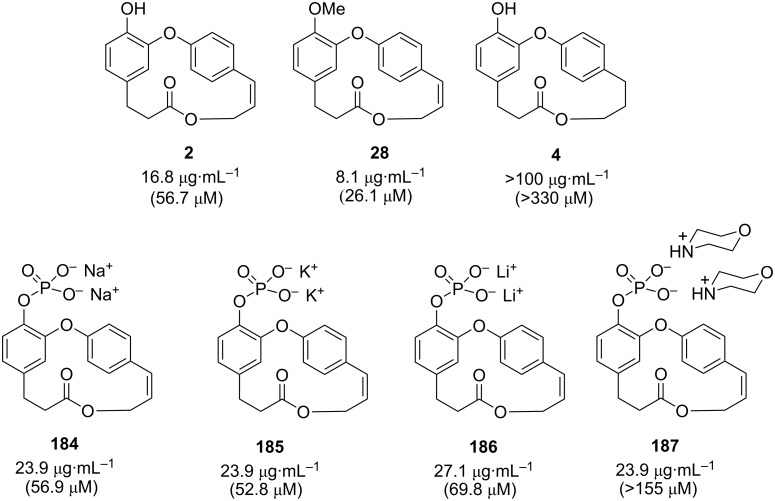
ED_50_ values of the combretastatin D family against murine P388 lymphocytic leukemia cell line (approximated values of molar concentration after conversion) [55].

However, when tested against the murine P388 lymphocytic leukemia cell line, salts **184–187** did not show enhanced inhibition of the cancer cell line growth compared to combretastatin D-2 (**2**) or the methylated congener **28**. Moreover, for structure–activity relationship studies, combretastatin D-4 (**4**) proved to be inactive indicating that the olefin was necessary for cancer cell growth inhibition. For salts **184**–**187**, the authors attributed the decrease in the activity to the lack of phosphatases necessary for the cleavage of the prodrug ester bond and needed to regenerate the drug in the isolated cancer cells (Figure 2).

The synthesized compounds were also evaluated in vitro against seven cancer cell lines and the concentration causing 50% cell growth inhibition (GI_50_) was determined. The results are collected in Table 4.

**Table 4:** Inhibition of cancer cell growth by combretastatin D-2 (**2**) and prodrug salts [55].

	GI_50_ mg·mL^−1^ (molar concentration)^a^					

	pancreas	breast	brain	lung	colon	prostate

**2**	4.8 (16.2 μM)	6.6 (22.3 μM)	4.7 (15.9 μM)	6.0 (20.2 μM)	>10 (33.7 μM)	2.7 (9.1 μM)
**28**	7.3 (23.5 μM)	0.83 (2.7 μM)	2.2 (7.1 μM)	6.6 (21.3 μM)	9.4 (30.3 μM)	5.6 (18 μM)
**184**	22.1 (52.6 μM)	30.5 (72.6 μM)	36.3 (86.4 μM)	13.3 (31.6 μM)	25.5 (60.7 μM)	18.2 (43.3 μM)
**185**	40.1 (88.6 μM)	34.5 (76.2 μM)	45.2 (99.9 μM)	27.8 (61.4 μM)	40.6 (89.7 μM)	14.4 (31.2 μM)
**186**	35.9 (92.5 μM)	26.1 (67.2 μM)	37.9 (97.6 μM)	14.4 (31.1 μM)	27.5 (70.8 μM)	4.3 (11.1 μM)
**187**	>10 (>18 μM)	>10 (>18 μM)	>10 (>18 μM)	>10 (>18 μM)	>10 (>18 μM)	>10 (>18 μM)

^a^Approximated values of molar concentration after conversion.

Once again, the prodrugs **184**–**187** did not show increased inhibitory activity of cancer cell growth when compared with compounds **2** and **28**, due the same lack of phosphatase cited before.

It is worth to note that the mechanism of action for these compounds is attributed to their ability to interfere in the dynamics of tubulin, a protein involved in the formation of the cytoskeleton. After binding to tubulin, they act as stabilizing agents, allowing the formation of microtubules in the early stages, but preventing their disassembly in the final stages of cell division, thus leading to apoptosis [15,55,74] in a mechanism similar to taxol^®^ [76]. However, it is worth to note that taxol^®^ is the most potent antitumor drug, showing GI_50_ ranging from pmol to nmol against different types of cancer cell lines [77].

When combretastatins D-2, D-4, and some synthetic intermediates were evaluated against *Candida albicans* (ATCC 90028), *Cryptococcus neoformans* (ATCC 90112), *Micrococcus luteus* (Presque Isle 456), *Staphylococcus aureus* (ATCC 29213), *Streptococcus pneumoniae* (ATCC 6303), *Escherichia coli* (ATCC 25922), *Stenotrophomonas maltophilia* (ATCC 13637), *Enterobacter cloacae* (ATCC 13047), *Enterococcus faecalis* (ATCC 29212), and *Neisseria gonorrhoeae* (ATCC 49226) no significant antibacterial or antifungal activities were observed [55].

Ponnapalli and co-workers tested in an agar diffusion assay the isolated compounds corniculatolide A (**4**), 11-*O*-methylcorniculatolide A (**5**), 12-hydroxy-11-*O*-methylcorniculatolide A (**6**), isocorniculatolide A (**7**), and 11-*O*-methylisocorniculatolide A (**8**) against the bacteria *Staphylococcus aureus*, *Bacillus subtilis*, *Micrococcus luteus*, *Escherichia coli*, *Klebsiella pneumoniae*, and the fungi *Aspergillus niger*, *Aspergillus terreus*, and *Aspergillus flavus*. Unfortunately, all compounds were found to be inactive [14].

**Figure 3:**
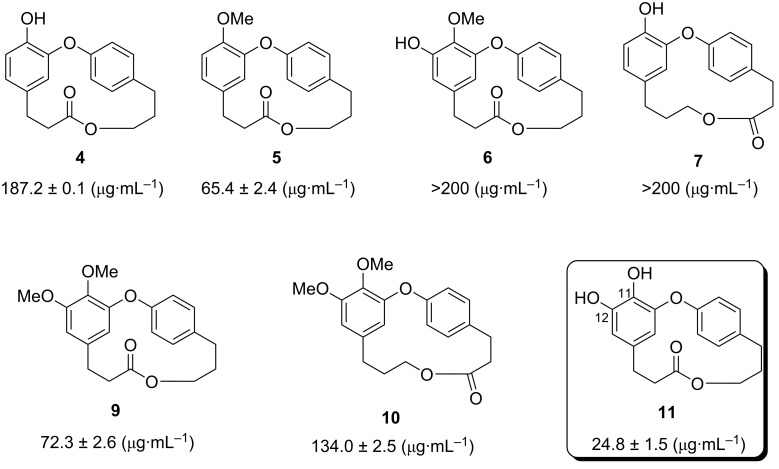
IC_50_ of compounds against α-glucosidase [19].

The use of compounds capable to delay glucose absorption by inhibiting the associated enzymes such as α-glucosidase, is one of the effective therapeutic methods in diabetes mellitus treatment [78]. Thus, Olanipekun and co-workers [19] evaluated the isolated corniculatolides and isocorniculatolides in their work against α-glucosidase from *Saccharomyces cerevisiae*. From the tested compounds, corniculatolide C (**11**) exhibited the most potent AGH inhibitory activity with an IC_50_ of 24.8 μM when compared with the antidiabetic drug acarbose (IC_50_ 12.2 ± 2.2 μM), while compounds **6** and **7** showed no inhibitory activity (IC_50_ > 200 μM) (Figure 3). The authors suggested that the presence of hydroxy substituents at C-11 and C-12 played a significant role in the α-glucosidase inhibition [19].

The cytotoxicity of the aforementioned compounds was also evaluated using the sulforhodamine B (SRB) assay against MIAPaCa-2, DU145, MCF-7, and HTC-116 human cancer cell lines. The compounds were tested in five different concentrations (ranging from 1 to 100 μM) and showed no in vitro cytotoxicity.

However, compound **10** was found to possess anti-inflammatory activity. It showed effects on LPS-induced activation of NF-κB and COX-2 similar to the Bay 11-7082 molecule in the intestinal epithelial cell line IEC-6. In addition, inhibition of mRNA expression of TNF-α, IL-1β, and IL-6 was observed, which means that this compound has potential pharmacological application towards inflammatory diseases such inflammatory bowel disease [71].

## Conclusion

A variety of efforts over the past years involving the synthesis of cyclic diaryl ether heptanoids (DAEHs), particularly combretastatins D series, corniculatolides, and isocorniculatolides have been illustrated. These efforts demonstrate the application of classic methodologies and provide new insights for newer methodologies. Despite the advances made, some synthetic challenges such as better yields for macrolactonization reactions, reduction in the number of synthetic steps, and selective deprotection of certain groups still persist. Thus, the development of more efficient and scalable strategies for further biological studies remains highly desirable. The pharmacological potential of these compounds also requires further studies since most of them showed activity on a micromolar scale in in vitro assays, with the compounds containing the *cis* double bond being the most active. However, the use of computational tools and new assay technologies for high throughput screening (HTS) could lead to the discovery of new analogues with more potent activities. Moreover, the study on the application of these compounds to neglected tropical diseases (NTDs), which include Chagas disease, leishmaniasis, and human African trypanosomiasis (HAT) has not yet been performed.
